# Formal and informal care use before, during, and after detection of cognitive impairment and dementia: A population-based matched study

**DOI:** 10.1177/13872877251350525

**Published:** 2025-06-23

**Authors:** Sakura Sakakibara, Abigail Dove, Jie Guo, Giulia Grande, Ulrika Akenine, Britt-Marie Sjölund, Janne Agerholm, Erika J Laukka, Amaia Calderon-Larrañaga, Weili Xu

**Affiliations:** 1 Aging Research Center, Department of Neurobiology, Care Sciences and Society, Karolinska Institutet, Stockholm, Sweden; 2 Stockholm Gerontology Research Centre, Stockholm, Sweden; 3 Division of Clinical Geriatrics, Center for Alzheimer Research, Department of Neurobiology, Care Sciences and Society, Karolinska Institutet, Stockholm, Sweden; 4 Faculty of Health and Occupational Studies, Department of Health and Caring Sciences, University of Gävle, Gävle, Sweden

**Keywords:** Alzheimer's disease, cognitive impairment, dementia, informal care, matched-pair analysis, social care

## Abstract

**Background:**

Dementia is linked to increased care use, but formal and informal care use throughout the dementia journey remains unclear.

**Objective:**

To investigate care use before and after the detection of cognitive impairment, no dementia (CIND) and dementia and to identify care-related factors.

**Methods:**

Within a population-based study, we matched older adults (≥78 years) who developed CIND (n = 244) and dementia (n = 175) with cognitively intact participants to form CIND/intact (n = 732) and dementia/intact (n = 525) samples. Dementia was clinically diagnosed and CIND was determined through a neuropsychological battery. Formal (from public and private providers) and informal (provided by family and friends) care use was interviewed. Care-related factors included age, sex, education, living alone, chronic diseases, and social network. Data was analyzed using logistic regressions and linear mixed-effect models.

**Results:**

Compared to cognitively intact participants, those with CIND had increased care use 3 years after detection (odds ratio [OR] 2.30 and 2.63, 95% confidence interval [95%CI] 1.02–5.18 and 1.25–5.53) and those with dementia had greater care use over time (OR 2.01, 95% CI 1.20–3.38 to OR 13.58, 95% CI 4.46–41.34). People with CIND/dementia showed 6.3 to 32.3 h rapid increase in informal care hours. Older age, female, living alone, and chronic diseases further increased care use.

**Conclusions:**

Formal and informal care use during the progression of cognitive impairment begins to increase at the CIND stage, but only informal care hours continue to increase. The findings highlight the complex care needs of people with cognitive impairment and the importance of coordination of care.

## Introduction

Over 55 million people worldwide, including 150,000 in Sweden, have dementia, with prevalence projected to reach 139 million by 2050.^1,2^ This underscores the importance of implementing effective strategies to address the growing impact of dementia on individuals, families, and healthcare systems.

In Sweden, formal care—including health and social care provided by regions, municipalities, and private companies—can be both publicly and privately provided, however, it is largely tax-funded and publicly regulated.^3^ Most health care services are funded by and organized on a regional level, whereas home health care and social services are funded by and organized on a municipal level. One exception is Region Stockholm, where also home health care is funded by the region.^4^ For individuals living with dementia formal social care is the main form of publicly provided care services, in addition to institutional care.^5^ In addition, older adults may also receive informal care from friends, family, and voluntary caregivers, which constitutes an important complement to formal care.

Dementia is characterized by a decline in cognitive and functional abilities, leading to a substantial reliance on formal and informal care and, eventually, institutionalization.^4,6^ This increased care use is further elevated with worsening dementia severity.^7–10^ Previous studies conducted in European countries demonstrated that care hours are also greater among people with dementia.^8,9,11^ In particular, informal care contributes to a considerable amount of care for people with dementia, accounting for 140 to 300 h per month.^9,12,13^ However, dementia has a long prodromal period, and no studies to our knowledge have addressed formal and informal care use before and after the detection of cognitive impairment.

So far, several demographic and socioeconomic factors including older age, sex, education, and co-residency have been identified as factors associated with greater care use among older adults regardless of the existence of dementia.^13–17^ However, it remains unclear to what extent these factors contribute to increased care use among individuals with cognitive impairment and how care-related factors differ before and after cognitive impairment is detected.

In the present study, using data from a population-based cohort study, the Swedish National Study on Aging and Care in Kungsholmen (SNAC-K), we aimed to assess changes in usage and hours of formal and informal care before, during, and after the detection of cognitive impairment and further to identify predictors of care.

## Methods

### Study design and participants

SNAC-K is an ongoing population-based longitudinal study including people aged 60 years and older living at home or in institutions in Kungsholmen, in central Stockholm.^18^ A total of 5111 participants were randomly sampled from 11 age cohorts (60, 66, 72, 78, 81, 84, 87, 90, 93, 96, and 99 + years). Out of the 4590 eligible participants, 3363 participants (participation rate: 73.3%) underwent baseline examination between 2001 and 2004. The younger age cohorts (≤72 years) were followed every 6 years and the older age cohorts (≥78 years) were followed every 3 years given the higher attrition rate and more rapid changes in health expected in older age. At each wave, all participants underwent a comprehensive examination by trained physicians and nurses. Information on sociodemographic and lifestyle factors, medical conditions, care use, and cognitive function were collected through structured interviews and clinical examinations.^18^ The detailed protocol for data collection can be accessed on the SNAC-K website (https://www.snac-k.se/). In this study, we used data from baseline to the latest follow-up wave between 2016 and 2019.

In total, 1991 individuals from the older age cohorts (≥78 years) had follow-up data available every 3 years. From these, we excluded individuals who were institutionalized (n = 193) and had dementia at baseline (n = 102). We additionally excluded participants with missing information on cognitive status at baseline (n = 336), leaving 1360 individuals eligible for inclusion in the analysis.

Two samples were identified from this population, a cognitive impairment, no dementia (CIND)-control sample and a dementia-control sample. First, data collection and assessment waves in which CIND/dementia was detected were identified for participants with CIND/dementia as a detection wave. For cognitively intact participants, a detection wave was simulated by assigning them a random detection wave. This approach was based on a previously used method and was intended to reduce potential bias and minimize discrepancies between the CIND/dementia group and the cognitively normal group.^19,20^ Second, participants with CIND/dementia were matched in a 1:2 ratio (according to age and sex at the detection wave, as well as the maximum follow-up time) with cognitively intact participants using the nearest neighbor matching method.^21^ This resulted in a CIND-control sample with 732 participants (244 participants with CIND and 488 cognitively intact participants) and a dementia-control sample with 525 participants (175 participants with dementia and 350 cognitively intact participants) (Supplemental Figure 1).

A bidirectional timescale was used to ascertain care use. Year 0 was defined as the data collection and assessment wave when CIND or dementia was detected for participants with CIND/dementia and the randomly assigned detection wave for cognitively intact participants. Pre- and post-detection periods were defined as preceding (3 years before) and following (3 years after) data collection and assessment waves of CIND/dementia detection, respectively.

### Assessment of care use

Information on care use was collected through nurse interviews (Supplemental Table 1). When participants were unable to provide this information themselves, a proxy respondent, such as a family caregiver, answered on their behalf.

***Formal care*** was defined as home social and health care offered by public or private providers. This included support with basic activities of daily living (ADL) (i.e., dressing/undressing, eating, going to the bathroom, showering/bathing, washing self, and transferring from bed to chair) and instrumental ADL (IADL) (i.e., cooking, grocery shopping, other purchases, laundry, dishes, cleaning, managing finance, and telephone calls) provided by the municipalities, as well as home health care (e.g., injections, redressing, and intravenous therapy) provided by the region.

***Informal care*** encompasses support with basic ADL and IADL provided by family members, friends, neighbors, or volunteers.^9,22^

Hours of formal and informal care received were recorded as number of hours per month using the Resource Utilization in Dementia instrument.^23^

### Assessment of CIND

CIND is characterized by objective cognitive deficits in the absence of dementia.^24^ At baseline and each follow-up wave, participants underwent a comprehensive neuropsychological battery including seven tasks addressing five major cognitive domains: episodic memory (free recall), language (category and letter fluency), executive function (Trail Making Test-B), perceptual speed (digit cancelation and pattern comparison), and visuospatial abilities (mental rotation).^25^ The raw scores obtained from the individual cognitive tests were standardized into Z-scores based on the age-specific baseline means and standard deviations.^26^ Cognitive domains were defined by either averaging the Z-scores when more than one test was available or using individual test Z-scores. CIND was identified as scoring ≥1.5 SDs below the age-specific means in at least one of the five cognitive domains. The same procedure was used to identify CIND occurrence over the follow-up period, applying the same cut-offs as for the baseline. This assessment follows an established and standardized procedure widely used in the field.^24,26,27^

### Diagnosis of dementia

Dementia, including Alzheimer's disease and other types of dementia, was clinically diagnosed according to the *Diagnostic and Statistical Manual of Mental Disorders,* 4th edition (DSM-IV) revised criteria using a three-step procedure.^28^ First, the examining physician conducted a preliminary diagnosis based on a thorough assessment of the participant's physical, neurological, and cognitive status. Second, the reviewing physician made a secondary diagnosis based on computerized data from the medical examination. Third, in case of discrepancies between the two diagnoses, a senior neurologist independent of the data collection made the final decision.^29^ For participants who died during the follow-up, dementia status was verified using medical records and/or death certificates.

### Assessment of covariates

Educational attainment was defined as the maximum years of formal schooling and categorized as primary, secondary, or university. Living arrangement was dichotomized as living alone or living with someone. Chronic disease count was defined as a number of total chronic diseases from 60 chronic disease categories, as previously explained.^30^ Social network was defined based on 5 items related to social connection (marital status, living arrangement, number of children, frequency of contacts, and social network size) and 5 items related to social support (satisfaction with contacts, material support, psychological support, sense of affinity with family and friends, and being part of a group of friends) and categorized as low and moderate/high, as described previously.^31^

### Statistical analysis

Characteristics of the study participants were compared using t-tests for continuous variables and chi-square tests for categorical variables. Descriptive analyses were performed to outline the care use within each CIND/dementia group pre-, at the wave of, and post-detection.

Conditional logistic regression models were used to estimate odds ratios (OR) and 95% confidence intervals (CI) for the associations between CIND/dementia status and care use. Linear mixed-effects models were used to estimate the β coefficients and 95% CIs for changes in care hours from pre- to post-detection period according to CIND/dementia status. Random effect was applied for intercept and a random slope of follow-up time from pre- to post-detection period for each individual and matched pair, with unstructured covariance to account for between-subject and between-matched-pair validity. To explore the differences in annual change in care hours across CIND/dementia status, we estimated the interaction between CIND/dementia status and the follow-up time as a fixed effect. Both the conditional logistic regression models and the linear mixed-effects models were first basic adjusted for socio-demographic factors (age, sex, and educational attainment) and then further adjusted for living alone, multimorbidity, and social network. We also explored possible factors related to care use among people with cognitive impairment using logistic regression models. Univariate models included each of the following covariates separately: age, sex, educational attainment, living alone, chronic disease count, and social network. Basic adjusted models included age, sex, and educational attainment.

In sensitivity analysis, we repeated logistic regression analyses for CIND/dementia and care use and care-related factors by restricting the population to those who participated in all the observation periods (from year −3 to year 3) to account for the influence of attrition. Analyses for changes in care use were repeated using log-transformed care hours to assess the impact of the positively skewed care hours on the estimates.

All analyses were performed using Stata SE 16.0 (StataCorp, College Station, TX). All p-values were two-sided and statistical significance was defined as p < 0.05.

## Results

### Characteristics of the study population

In the CIND-control sample (n = 732), people with CIND were more likely to have lower educational attainment compared to cognitively intact participants (Table 1). In the dementia-control sample (n = 525), the incident dementia group was more likely to be older and have lower educational attainment and higher numbers of chronic diseases compared to the control group. Individuals included in our study population had more favorable profiles compared to those who were excluded (Supplemental Table 2).

**Table 1.** Characteristics of the study participants by matched cognitive impairment, no dementia (CIND)-control (n = 732) and dementia-control (n = 525) samples.

	CIND-control sample (n = 732)			Dementia-control sample (n = 525)		
Characteristics	Cognitively intact	CIND	*p*	Cognitively intact	Dementia	*p*
	(n = 488)	(n = 244)		(n = 350)	(n = 175)	
Age	85.9 (±5.1)	86.3 (±4.8)	0.31	86.2 (±4.7)	88.6 (±5.5)	<0.001
Female	295 (60.5%)	161 (66.0%)	0.15	225 (64.3%)	116 (66.3%)	0.65
Education			0.036			0.015
Elementary	74 (15.2%)	50 (20.5%)		52 (14.9%)	34 (19.4%)	
High school	244 (50.1%)	130 (53.3%)		180 (51.4%)	103 (58.9%)	
University	169 (34.7%)	64 (26.2%)		118 (33.7%)	38 (21.7%)	
Living alone	297 (61.0%)	164 (67.2%)	0.1	221 (63.1%)	116 (67.1%)	0.38
Disease count	6.8 (±3.4)	6.7 (±3.2)	0.75	6.5 (±3.4)	7.4 (±3.4)	0.004
Low social network	160 (34.0%)	76 (31.9%)	0.57	109 (31.4%)	62 (37.8%)	0.15

Data are presented as means ± standard deviations or number (proportion %)

CIND: cognitive impairment, no dementia.

Missing values (CIND-control; dementia-control): 1;2 in living alone, 24;14 in social network.

Since the matching did not completely reduce the differences in the characteristics between CIND/dementia and cognitively intact groups, confounders were adjusted in the main analyses.

### Description of care journey from pre- to post-detection of CIND/dementia

**Figure 1.**
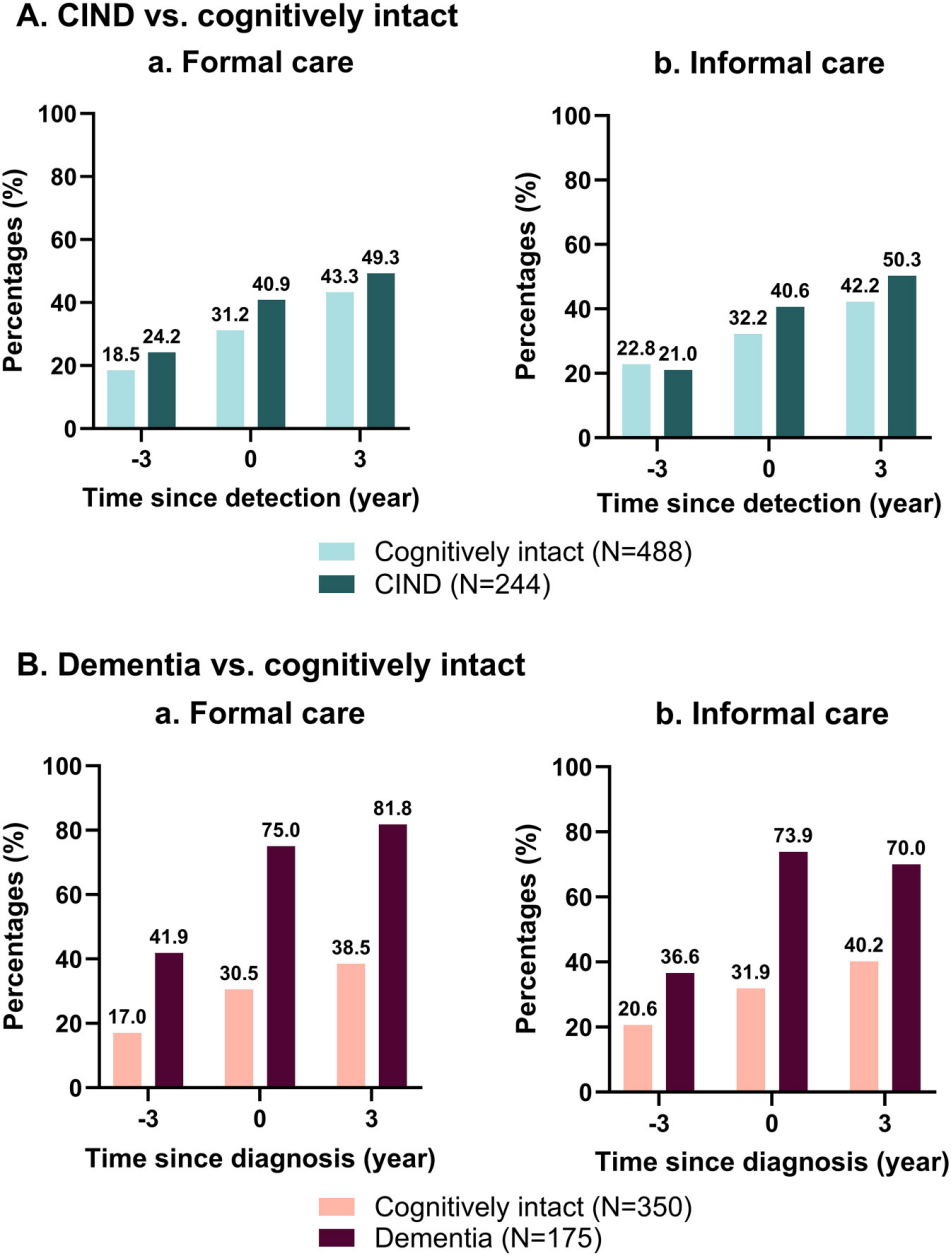
Proportion of care users pre-, at the wave of, and post-detection by cognitive impairment, no dementia (CIND)/dementia status.

In the CIND-control sample, formal and informal care use gradually increased from 3 years before to 3 years after detection. Formal care use increased by 24.8% in the cognitively intact participants and by 25.1% in the CIND participants, while informal care use increased by 19.4% and 29.3%, respectively (Figure 1).

CIND: cognitive impairment, no dementia.

Number of participants at year 3: 167;282 (CIND; cognitively intact), 60;197 (dementia; cognitively intact).

Within the dementia-control sample, formal care use increased by 21.5% and informal care use by 19.6% for cognitively intact participants. For those with incident dementia, formal care increased by 39.9% and informal care by 33.4%.

### Use of formal and informal care in people with cognitive impairment

Compared to the cognitively intact participants, participants with CIND were more likely to receive formal (OR 2.30, 95% CI 1.02–5.18) and informal care (OR 2.63, 95% CI 1.25–5.53) 3 years after the detection. Participants with incident dementia were more likely to receive formal (OR 2.80, 95% CI 1.57–5.01 to OR 13.58, 95% CI 4.46–41.34) and informal care (OR 2.01, 95% CI 1.20–3.38 to OR 5.82, 95% CI 2.72–12.43) throughout the follow-up period (Table 2).

**Table 2.** Odds ratios (OR) and 95% confidence intervals (CI) for care use in relation to cognitive impairment, no dementia (CIND) and dementia.

CIND-control sample					
Type of care use	Time to detection (year)	No. of subjects		OR (95% CI)	
		Cognitively intact (N = 488)	CIND (N = 244)	Model 1^a^	Model 2^b^
Formal care	−3	488	244	1.35 (0.89–2.05)	1.48 (0.95–2.33)
	0	488	244	1.27 (0.87–1.86)	1.35 (0.90–2.03)
	3	282	167	**2.16** (**1.00–4.66)**	**2.30** (**1.02–5.18)**
Informal care	−3	488	244	0.80 (0.53–1.19)	0.86 (0.55–1.34)
	0	488	244	1.31 (0.92–1.86)	1.32 (0.91–1.93)
	3	282	167	**1.98** (**1.07–3.66)**	**2.63** (**1.25–5.53)**

Dementia-control sample					
Type of care use	Time to detection (year)	No. of subjects		OR (95% CI)	
		Cognitively intact (N = 350)	Dementia (N = 175)	Model 1^a^	Model 2^b^
Formal care	−3	350	175	**2.51** (**1.50–4.22)**	**2.80** (**1.57–5.01)**
	0	350	175	**6.86** (**3.22–14.63)**	**8.27** (**3.52–19.43)**
	3	197	60	**12.01** (**4.15–34.75)**	**13.58** (**4.46–41.34)**
Informal care	−3	350	175	**1.68** (**1.04–2.69)**	**2.01** (**1.20–3.38)**
	0	350	175	**4.40** (**2.63–7.36)**	**4.82** (**2.76–8.41)**
	3	197	60	**5.32** (**2.59–10.91)**	**5.82** (**2.72–12.43)**

Bold type indicates statistical significance (p < 0.05).

^a^ Adjusted for age, sex, and educational attainment.

^b^ Further adjusted for living alone, disease count, and social network.

### Changes in hours of care use during the CIND/dementia journey

**Figure 2.**
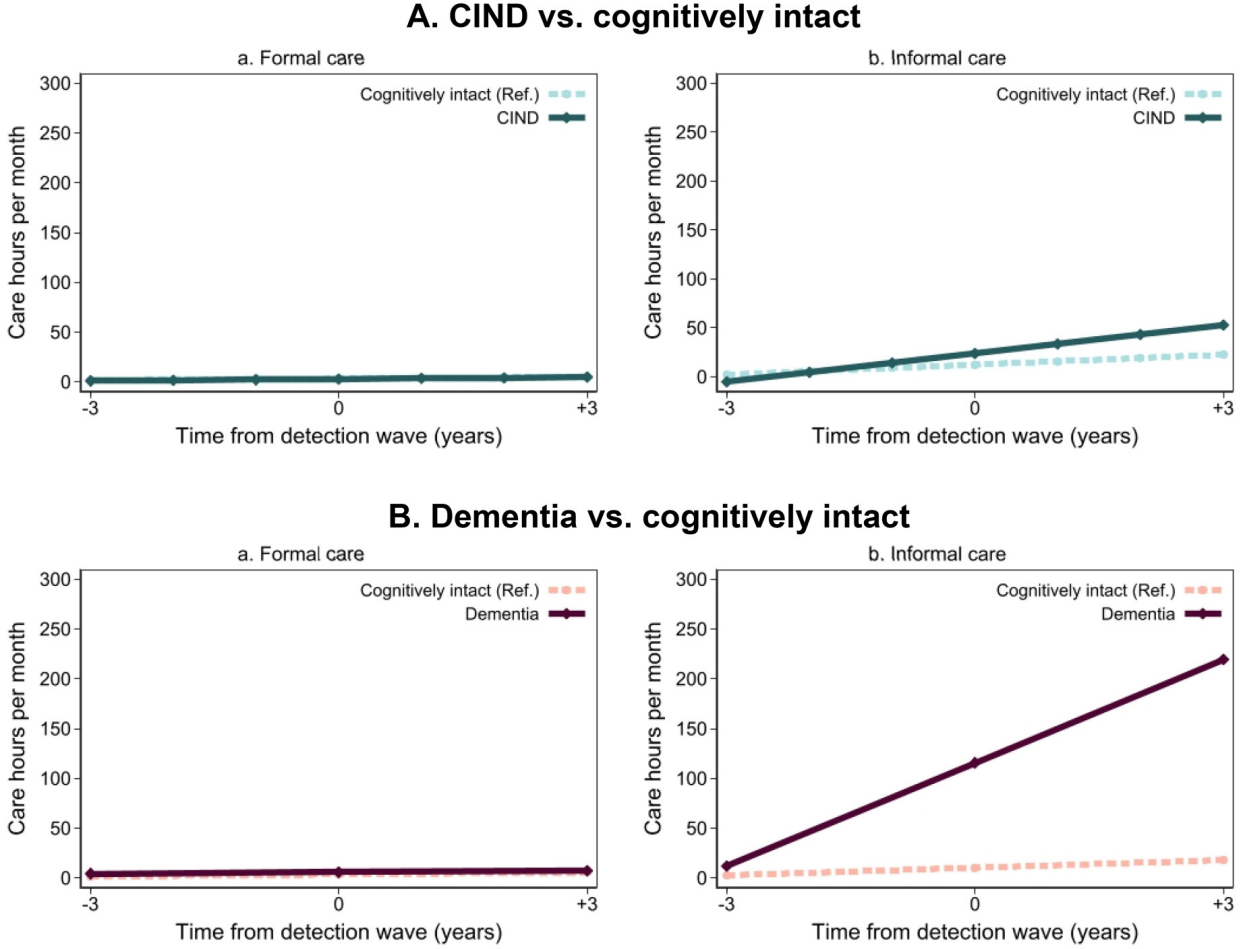
Trajectories of care hours by cognitive impairment, no dementia (CIND)/dementia status before 3 years to after 3 years of detection.

The annual changes in formal and informal care hours per month from pre- to post-detection period are shown in Figure 2 (Supplemental Table 3). Compared to cognitively intact controls, people with CIND showed a significant increase in the number hours of informal care received (β: 6.27, 95% CI 2.93, 9.60), although the trajectory of formal care use remained flat. A similar pattern was observed among dementia cases compared to controls, with an even steeper trajectory of increased hours of informal care received (β: 32.25, 95% CI 25.46, 39.05).

Linear mixed-effect models multi adjusted for age, sex, educational attainment, living alone, disease count, and social network. β coefficients and 95% confidence intervals are available in Supplemental Table 3.

CIND: cognitive impairment, no dementia.

### Factors related to care use

**Figure 3.**
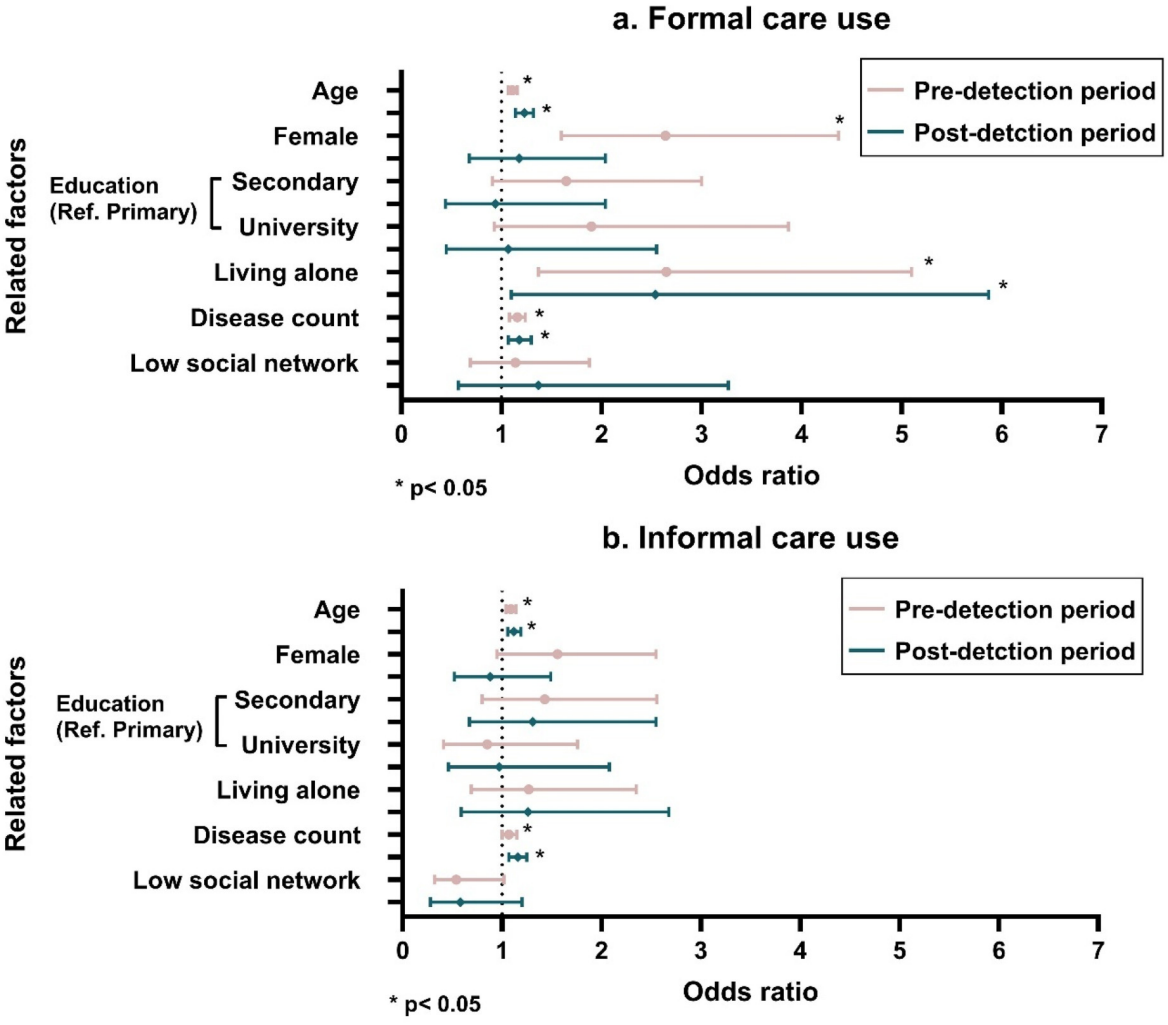
Factors related to formal and informal care use in people with cognitive impairment, no dementia (CIND) or dementia during pre- and post-detection periods among those who had information on care use for both pre-and post-detection (n = 387).

Factors related to formal and informal care use in pre- and post-detection period among people with CIND or dementia (n = 387) are shown in Figure 3 (Supplemental Table 4). Prior to CIND/dementia detection, participants who were older (OR 1.11, 95% CI 1.07–1.16), female (OR 2.64, 95% CI 1.60–4.37), live alone (OR 2.65, 95% CI 1.37–5.10), and have more chronic diseases (OR 1.16, 95% CI 1.08–1.24) were more likely to use formal care. These factors other than sex were also significantly associated with formal care use in the post-detection period. For informal care use, older age (OR 1.09, 95% CI 1.04–1.14) and chronic disease (OR 1.07, 1.00–1.15) were associated with increased care use in the pre-detection period, as well as the post-detection period.

Models were adjusted for age, sex, and educational attainment when available. Underlying odds ratios and 95% confidence intervals are presented in Supplemental Table 4.

### Sensitivity analyses

Similar results were observed in the overall direction of the estimates after: (1) restricting the population to those who participated in the follow-up assessments both pre- and post-detection periods (Supplemental Tables 5 and 6) and (2) using log-transformed care hours, given the skewed distribution (Supplemental Table 7).

## Discussion

In this population-based study, we found that: (1) compared to cognitively intact people, those with CIND had higher formal and informal care use 3 years after the CIND detection and those with dementia had greater formal and informal care use from pre- to post-diagnosis period; (2) older adults affected CIND or dementia use more hours of informal care than cognitively intact individuals; and (3) advanced age, female sex, living alone, and somatic disease further increase the care use among people affected by CIND or dementia.

In Sweden, formal care organized at the municipal level is widely available, and most of the costs are covered by taxes.^32,33^ Formal care is viewed quite favorably,^34,35^ and policies such as the Social Service Act have encouraged older adults to age in place while receiving home care rather than moving to an institution.^36^ To this end, home care use in Sweden has expanded over the past few decades.^15^ However, the formal care system struggles to keep up with population aging and the ever-growing number of older adults in need of extensive care at home.^16,37^ Informal care can fill this gap but is not always accessible for some older adults. A large proportion of older people in urban areas live alone and may not have family members or friends nearby.^22^ Additionally, working-age individuals may have limited time to devote to caring for older relatives.^38^

Individuals with CIND had two to three times greater formal and informal care use 3 years after the detection, and those with dementia had two to fourteen times higher formal and informal care use from the pre- to post-diagnosis compared to cognitively intact participants. Observed increased care use before formal dementia diagnosis is consistent with previous research showing increased care use among people at the earliest symptomatic stages of dementia compared to those cognitively intact.^9^ An increase in care needs among older individuals may serve as an indicator of the presence of CIND, facilitating earlier detection of cognitive impairment. Assessing care use among people with CIND, a marker of prodromal dementia, is important due to its higher incidence than dementia and the higher risk of progression to dementia.^39,40^ Similarly, increased formal and informal care use provides a valuable opportunity for early detection of dementia and early intervention for person-centered care planning for people with dementia and their informal caregivers. We observed greater formal care use after dementia diagnosis among people with dementia compared to cognitively intact individuals. This increase may be attributed to the progression of the disease, leading to greater care needs,^41^ as well as the formal diagnosis itself, which may have facilitated access to and use of formal care services.^42^

We found a faster increase in the hours of informal care received among older adults with CIND/dementia. Previous studies have reported a wide range of estimates for informal care usage from 140 to 300 h in a month for people with dementia^9,11–13,43^ and 120 h of informal care per month for people with CIND.^40^ However, these studies either used a cross-sectional design or did not specify the time elapsed since the dementia/CIND detection. Meanwhile, formal care hours remained constant over time in the present study. A possible explanation could be that there are potential barriers hindering access to an adequate amount of formal care services, or limitations regarding the amount and type of formal care individuals can receive.^44,45^ It is particularly crucial to accurately quantify care hours over time, especially considering that care dependency increases as dementia progresses.

During the pre-detection period, advanced age, living alone, and chronic diseases further increased both formal and informal care use among people with cognitive impairment. It is not surprising that care dependency increases in older age due to functional impairment.^46^ Women are generally more likely to become widowed and have less access to informal care within home, leading to formal care use.^47^ Older adults who live alone tend to require formal care due to the absence of household informal caregivers.^48,49^ Having multiple chronic diseases affects health and functional outcomes to varying degrees depending on the combination and interaction of diseases.^50,51^ After the detection of CIND/dementia, these associations remained, except for sex in formal care use. This suggests that older adults with these characteristics have higher care needs even before cognitive impairment is detected, with continued demand as the condition progresses. Identifying populations with increased care needs provides essential evidence for person-centered care planning.

While our findings revealed the patterns of formal and informal care use among older adults with cognitive impairment, there is an urgent need for further investigation into the economic evaluation and qualitative aspects of care experience in this population. Conducting comprehensive economic evaluations can provide valuable insight into planning and distributing formal care resources to keep up with the growing number of older adults in need of care. By integrating qualitative data on the experiences of individual care users and providers with quantitative data, more holistic and person-centered care can be developed that are tailored to the unique needs and preferences of individuals on trajectories of cognitive decline.

### Strengths and limitations

Strengths of this study lie in its use of a population-based cohort study with a comprehensive data collection procedure, including the ascertainment of CIND/dementia and care use over time and the integration of data from multiple sources. However, several limitations should be acknowledged. First, the statistical power might have been limited due to the reduction of the sample size after matching, making it challenging to detect small yet significant differences between groups. Second, the reporting of care use is subject to potential recall bias among people with cognitive impairment. Especially, reports of informal care use and the amount of care received may have been influenced by whether the participant or their proxy (potential caregivers) provided the information. However, the interview was carefully designed to systematically inquire about care use. Third, information on care and cognitive status was collected on a three-year cycle due to the nature of the SNAC-K study design. Therefore, some cases of CIND or dementia may have been detected in a delayed manner, potentially contributing to an underestimation of the reported associations. Moreover, changes in care use between waves could not be captured, limiting the ability to track short-term fluctuations. Additionally, the generalizability of these findings—which reflect an affluent, urban, highly educated population—may be limited. Finally, potential residual confounding due to unmeasured factors cannot be entirely ruled out.

### Conclusion

In this population-based study, people with dementia had increased formal and informal care use 3 years before to 3 years after the diagnosis compared to cognitively intact people. People with CIND had greater formal and informal care use 3 years after CIND detection. Older adults affected by CIND or dementia had a rapid increase in informal care hours compared to cognitively intact individuals. Moreover, advanced age, female sex, living alone, and chronic diseases further increase the care use among people with cognitive impairment. The findings not only highlight an increase in care use as a potential early warning sign of CIND/dementia, but also call attention to the complex care needs of older people before, during, and after the emergence of cognitive impairment. In an aging population with a growing number of people with CIND/dementia, there is a need to consider how formal and informal care is used for future care planning and adjustment.

## Supplemental Material

sj-docx-1-alz-10.1177_13872877251350525 - Supplemental material for Formal and informal care use before, during, and after detection of cognitive impairment and dementia: A population-based matched study Supplemental material, sj-docx-1-alz-10.1177_13872877251350525 for Formal and informal care use before, during, and after detection of cognitive impairment and dementia: A population-based matched study by Sakura Sakakibara, Abigail Dove, Jie Guo, Giulia Grande, Ulrika Akenine, Britt-Marie Sjölund, Janne Agerholm, Erika J Laukka, Amaia Calderon-Larrañaga and Weili Xu in Journal of Alzheimer's Disease
