# Supplementary material for: Formal and informal care use before, during, and after detection of cognitive impairment and dementia: A population-based matched study
Source: J Alzheimers Dis. 2025 Jun 23;106(3):1010–20. doi: 10.1177/13872877251350525 (PMC12284328; doi:10.1177/13872877251350525)
Supplement: sj-docx-1-alz-10.1177_13872877251350525 - Supplemental material for Formal and informal care use before, during, and after detection of cognitive impairment and dementia: A population-based matched study [file sj-docx-1-alz-10.1177_13872877251350525.docx]

**Supplemental Material**

**Formal and informal care use before, during, and after detection of cognitive impairment and dementia: A population-based matched study**

**Supplemental Table 1.** Questionnaire for the assessment of formal and informal care use

| **Care type** | **Questionnaire** |
| --- | --- |
| **Formal care** | Do you get any sort of service (household chores), personal care, or medical care assistance from municipal or regional authorities? |
|  | Do you get any sort of service assistance (e.g., cooking, grocery shopping, other purchases, laundry, dishes, cleaning, managing finances, telephone calls, riding/driving car, other transportation) from municipal authorities? |
|  | Do you get any sort of personal care assistance? (e.g., getting dressed/undressed, eating, going to the bathroom, showering/bathing, washing self, moving around) |
|  | Do you get any sort of health care assistance in your home? (e.g., injections, redressing, IV, compression stockings, care of urinary catheter) |
| **Informal care** | Do you receive any assistance from relatives, friends, neighbors or volunteer/non-profit organizations regarding service or care? |
|  | In the last month, did you receive any service assistance (e.g., cooking, grocery shopping, other purchases, laundry, dishes, cleaning, finances, telephone calls, ride/drive car, other transportation)? |
|  | In the last month, did you receive any assistance with personal care (e.g., eating, dressing, undressing, toilet needs, showering/bathing, washing, moving around)? |

**Supplemental Table 2.** SNAC-K baseline characteristics by missingness of information on cognitive status among participants without dementia diagnoses (n=1696).

| **Characteristics** | **Cognitive status available**  **(n=1,341)** | **Missing on cognitive status**  **(n=345)** | ***p*** |
| --- | --- | --- | --- |
| Age, y | 80.3 (±6.5) | 85.0 (±7.2) | <0.001 |
| Female | 901 (66.3%) | 256 (76.2%) | <0.001 |
| Education |  |  | <0.001 |
| Elementary | 278 (20.4%) | 100 (30.4%) |  |
| High school | 731 (53.8%) | 191 (58.1%) |  |
| University | 351 (25.8%) | 38 (11.6%) |  |
| Living alone | 842 (62.1%) | 251 (75.8%) | <0.001 |
| Number of ADL limitations | 0.1 (0.3) | 0.3 (0.9) | <0.001 |
| Disease count | 4.6 (2.3%) | 5.5 (2.8%) | <0.001 |
| Low social network | 439 (38.7%) | 31 (63.3%) | <0.001 |

Data are presented as means ± standard deviations or number (proportion %)

ADL: activities of daily living

Missing variables: 9 in living alone, 9 in number of ADL limitations, 512 in social network.

**Supplemental Table 3.** β-coefficients and 95% confidence intervals (CI) for the association between hours of care and cognitive impairment, no dementia (CIND)/dementia status.

| **Care use hours** | **CIND/dementia × time** | **n** | **Model 1^a^**  **β (95% CI)^c^** | **Model 2^b^**  **β (95% CI)^c^** |  |
| --- | --- | --- | --- | --- | --- |
| **CIND-control sample** | | | | | |
| Formal care hours | No CIND × time | 488 | Reference | Reference |  |
|  | CIND × time | 244 | 0.07 (-0.41, 0.56) | 0.08 (-0.40, 0.56) |  |
| Informal care hours | No CIND × time | 488 | Reference | Reference |  |
|  | CIND × time | 244 | **6.28 (2.95, 9.62)** | **6.27 (2.93, 9.60)** |  |
| **Dementia-control sample** | | | | | |
| Formal care hours | No dementia × time | 350 | Reference | Reference |  |
|  | Dementia × time | 175 | -0.29 (-1.35, 0.77) | -0.28 (-1.32, 0.77) |  |
| Informal care hours | No dementia × time | 350 | Reference | Reference |  |
|  | Dementia × time | 175 | **32.15 (25.36, 38.96)** | **32.25 (25.46, 39.05)** |  |

^a^ Adjusted for age, sex, and educational attainment.

^b^ Further adjusted for living alone, disease count, and social network.

^c^ β-coefficients representing interactions between cognitive status and follow-up time.

**Supplemental Table 4.** Factors related to formal and informal care use in people with cognitive impairment, no dementia (CIND) or dementia during pre- and post-detection periods (n=387)

| **Characteristics** | |  | **Pre-detection period** | | |  | **Post-detection period** | | | |
| --- | --- | --- | --- | --- | --- | --- | --- | --- | --- | --- |
|  |  | **No. of subjects** | **Care use (%)** | **Model 1^a^**  **OR (95% CI)** | **Model 2^b^**  **OR (95% CI)** |  | **No. of subjects** | **Care use (%)** | **Model 1^a^**  **OR (95% CI)** | **Model 2^b^**  **OR (95% CI)** |
| **Formal care** |  |  |  |  |  |  |  |  |  |  |
| Age |  | 387 | 125 (32.3) | **1.11 (1.07-1.16)** |  |  | 195 | 81 (41.5) | **1.23 (1.14-1.32)** |  |
| Sex | Male | 129 | 25 (19.4) | Reference |  |  | 59 | 24 (40.7) | Reference |  |
|  | Female | 258 | 100 (38.8) | **2.64 (1.60-4.37)** |  |  | 136 | 57 (41.9) | 1.18 (0.68-2.04) |  |
| Education | Primary | 82 | 24 (29.3) | Reference | Reference |  | 36 | 15 (41.7) | Reference | Reference |
|  | Secondary | 209 | 72 (34.4) | 1.29 (0.74-2.25) | 1.65 (0.91-3.00)^c^ |  | 103 | 40 (38.8) | 0.78 (0.38-1.59) | 0.94 (0.44-2.04)^c^ |
|  | University | 96 | 29 (30.2) | 1.06 (0.56-2.03) | 1.90 (0.93-3.87)^c^ |  | 56 | 26 (46.4) | 0.68 (0.31-1.52) | 1.07 (0.45-2.55)^c^ |
| Living alone | No | 120 | 20 (16.7) | Reference | Reference |  | 50 | 18 (36.0) | Reference | Reference |
|  | Yes | 267 | 105 (39.3) | **3.30 (1.92-5.66)** | **2.65 (1.37-5.10)** |  | 145 | 63 (43.4) | **3.19 (1.65-6.19)** | **2.54 (1.10-5.87)** |
| Disease count | No | 387 | 125 (32.3) | **1.15 (1.07-1.23)** | **1.16 (1.08-1.24)** |  | 195 | 81 (41.5) | **1.19 (1.09-1.29)** | **1.18 (1.07-1.30)** |
| Low social network | No | 241 | 71 (29.5) | Reference | Reference |  | 133 | 57 (42.9) | Reference | Reference |
|  | Yes | 130 | 46 (35.4) | 1.31 (0.83-2.07) | 1.14 (0.69-1.88) |  | 55 | 23 (41.8) | **1.86 (1.00-3.44)** | 1.37 (0.57-3.27) |
| **Informal care** |  |  |  |  |  |  |  |  |  |  |
| Age |  | 387 | 109 (28.2) | **1.09 (1.04-1.14)** |  |  | 195 | 89 (45.6) | **1.12 (1.06-1.19)** |  |
| Sex | Male | 129 | 29 (22.5) | Reference |  |  | 59 | 27 (45.8) | Reference |  |
|  | Female | 258 | 80 (31.0) | 1.56 (0.95-2.55) |  |  | 136 | 62 (45.6) | 0.88 (0.52-1.49) |  |
| Education | Primary | 82 | 23 (28.0) | Reference | Reference |  | 36 | 13 (36.1) | Reference | Reference |
|  | Secondary | 209 | 67 (32.1) | 1.22 (0.69-2.14) | 1.43 (0.80-2.56)^c^ |  | 103 | 53 (51.5) | 1.12 (0.59-2.12) | 1.31 (0.67-2.55)^c^ |
|  | University | 96 | 19 (19.8) | 0.63 (0.32-1.27) | 0.85 (0.41-1.76)^c^ |  | 56 | 23 (41.1) | 0.76 (0.37-1.55) | 0.97 (0.46-2.08)^c^ |
| Living alone | No | 120 | 27 (22.5) | Reference | Reference |  | 50 | 24 (48.0) | Reference | Reference |
|  | Yes | 267 | 82 (30.7) | 1.54 (0.93-2.53) | 1.27 (0.69-2.35) |  | 145 | 65 (44.8) | 1.44 (0.77-2.69) | 1.26 (0.59-2.68) |
| Disease count | No | 387 | 109 (28.2) | **1.10 (1.03-1.17)** | **1.07 (1.00-1.15)** |  | 195 | 89 (45.6) | **1.16 (1.08-1.25)** | **1.16 (1.07-1.25)** |
| Low social network | No | 241 | 77 (32.0) | Reference | Reference |  | 133 | 66 (49.6) | Reference | Reference |
|  | Yes | 130 | 30 (23.1) | 0.65 (0.40-1.05) | 0.54 (0.32-1.02) |  | 55 | 20 (36.4) | 1.07 (0.63-1.81) | 0.58 (0.28-1.20) |

^a^ Univariate model. ^b^ Adjusted for age, sex, and educational attainment. ^c^ Adjusted for age and sex.

**Supplemental Table 5.** Odds ratios (OR) and 95% confidence intervals (CI) for care use in relation to cognitive impairment, no dementia (CIND) and dementia status among those who participated all the waves (-3, 0, +3).

| **CIND-control sample** | | | | | | | |
| --- | --- | --- | --- | --- | --- | --- | --- |
| **Type of care use** | **Time to detection (y)** | | **Cognitively intact**  **(N=282)** | **CIND**  **(N=167)** | **Model 1^a^** | **Model 2 ^b^** |  |
|  |  |  | **Care use n (%)** | **Care use n (%)** | **OR (95% CI)** | **OR (95% CI)** |  |
| Formal care use | -3 | | 35 (12.4) | 34 (20.4) | 2.09 (0.95-4.61) | 2.25 (0.96-5.29) |  |
|  | 0 | | 65 (23.5) | 55 (32.9) | 1.37 (0.76-2.47) | 1.42 (0.75-2.68) |  |
|  | 3 | | 89 (42.2) | 72 (49.3) | **2.16 (1.00-4.66)** | **2.30 (1.02-5.18)** |  |
| Informal care use | -3 | | 44 (15.6) | 32 (19.3) | 0.96 (0.49-1.85) | 1.23 (0.54-2.78) |  |
|  | 0 | | 66 (23.4) | 57 (34.1) | **1.77 (1.01-3.10)** | **1.94 (1.00-3.75)** |  |
|  | 3 | | 91 (41.2) | 77 (49.7) | **1.98 (1.07-3.66)** | **2.63 (1.25-5.55)** |  |
| **Dementia-control sample** | | | | | | | |
|  | |  | **Cognitively intact**  **(N=197)** | **Dementia**  **(N=60)** | **Model 1^a^** | **Model 2 ^b^** |  |
| **Type of care use** | **Time to detection (y)** | | **Care use n (%)** | **Care use n (%)** | **OR (95% CI)** | **OR (95% CI)** |  |
| Formal care use | -3 | | 29 (14.7) | 17 (28.3) | 1.80 (0.86-3.76) | 1.81 (0.84-3.89) |  |
|  | 0 | | 51 (26.3) | 25 (67.6) | **6.49 (2.80-15.04)** | **8.46 (3.36-21.26)** |  |
|  | 3 | | 71 (38.2) | 18 (81.8) | **12.01 (4.15-34.75)** | **13.58 (4.46-41.34)** |  |
| Informal care use | -3 | | 30 (15.2) | 12 (20.0) | 1.09 (0.50-2.40) | 1.18 (0.52-2.66) |  |
|  | 0 | | 52 (26.4) | 32 (65.3) | **4.54 (2.25-9.17)** | **5.06 (2.42-10.59)** |  |
|  | 3 | | 77 (39.9) | 28 (70.0) | **5.32 (2.59-10.91)** | **5.82 (2.72-12.43)** |  |

^a^ Adjusted for age, sex, and educational attainment.

^b^ Further adjusted for living alone, disease count, and social network.

**Supplemental Table 6.** Factors related to formal and informal care use in people with cognitive impairment, no dementia (CIND) or dementia during pre- and post-detection periods among those who had information on care use for both pre-and post-detection (n=159)

| **Characteristics** | |  | **Pre-detection period** | | |  | **Post-detection period** | | | |
| --- | --- | --- | --- | --- | --- | --- | --- | --- | --- | --- |
|  |  | **No. of subjects** | **Care use (%)** | **Model 1^a^**  **OR (95% CI)** | **Model 2^b^**  **OR (95% CI)** |  | **No. of subjects** | **Care use (%)** | **Model 1^a^**  **OR (95% CI)** | **Model 2^b^**  **OR (95% CI)** |
| **Formal care** |  |  |  |  |  |  |  |  |  |  |
| Age |  | 195 | 45 (23.1) | **1.11 (1.04-1.19)** |  |  | 195 | 81 (41.5) | **1.18 (1.08-1.28)** |  |
| Sex | Male | 59 | 6 (10.2) | Reference |  |  | 59 | 24 (40.7) | Reference |  |
|  | Female | 136 | 39 (28.7) | **3.55 (1.41-8.93)** |  |  | 136 | 57 (41.9) | 1.47 (0.74-2.91) |  |
| Education | Primary | 36 | 5 (13.9) | Reference | Reference |  | 36 | 15 (41.7) | Reference | Reference |
|  | Secondary | 103 | 29 (28.2) | 2.43 (0.86-6.86) | 2.78 (0.95-8.16)^c^ |  | 103 | 40 (38.8) | 0.86 (0.36-2.09) | 0.85 (0.33-2.19)^c^ |
|  | University | 56 | 11 (19.6) | 1.52 (0.48-4.80) | 2.13 (0.64-7.11)^c^ |  | 56 | 26 (46.4) | 1.09 (0.42-2.87) | 1.34 (0.47-3.79)^c^ |
| Living alone | No | 63 | 7 (11.1) | Reference | Reference |  | 50 | 18 (36.0) | Reference | Reference |
|  | Yes | 132 | 38 (28.8) | **3.23 (1.35-7.73)** | 2.22 (0.77-6.42) |  | 145 | 63 (43.4) | **2.76 (1.36-5.62)** | 2.36 (0.97-5.78) |
| Disease count | No | 195 | 45 (23.1) | **1.15 (1.04-1.26)** | **1.14 (1.03-1.27)** |  | 195 | 81 (41.5) | **1.15 (1.05-1.25)** | **1.17 (1.05-1.29)** |
| Low social network | No | 133 | 31 (23.3) | Reference | Reference |  | 133 | 57 (42.9) | Reference | Reference |
|  | Yes | 55 | 11 (20.0) | 0.82 (0.38-1.78) | 0.74 (0.31-1.74) |  | 55 | 23 (41.8) | 1.26 (0.60-2.65) | 1.36 (0.54-3.39) |
| **Informal care** |  |  |  |  |  |  |  |  |  |  |
| Age |  | 195 | 38 (19.5) | **1.10 (1.03-1.19)** |  |  | 195 | 89 (45.6) | 1.05 (0.99-1.13) |  |
| Sex | Male | 59 | 6 (10.2) | Reference |  |  | 59 | 27 (45.8) | Reference |  |
|  | Female | 136 | 32 (23.5) | **2.74 (1.08-6.97)** |  |  | 136 | 62 (45.6) | 1.07 (0.56-2.04) |  |
| Education | Primary | 36 | 3 (8.3) | Reference | Reference |  | 36 | 13 (36.1) | Reference | Reference |
|  | Secondary | 103 | 25 (24.3) | **3.57 (1.01-12.65)** | **4.04 (1.11-14.72)^c^** |  | 103 | 53 (51.5) | 1.69 (0.74-3.87) | 1.73 (0.75-4.02)^c^ |
|  | University | 56 | 10 (17.9) | 2.39 (0.61-9.37) | 3.25 (0.79-13.29)^c^ |  | 56 | 23 (41.1) | 1.25 (0.50-3.15) | 1.35 (0.51-1.95)^c^ |
| Living alone | No | 63 | 5 (7.9) | Reference | Reference |  | 50 | 24 (48.0) | Reference | Reference |
|  | Yes | 132 | 33 (25.0) | **3.91 (1.44-10.57)** | 3.22 (0.99-10.48) |  | 145 | 65 (44.8) | 1.19 (0.61-2.31) | 1.10 (0.49-2.44) |
| Disease count | No | 195 | 38 (19.5) | **1.11 (1.01-1.23)** | **1.11 (1.00-1.24)** |  | 195 | 89 (45.6) | **1.13 (1.04-1.22)** | **1.14 (1.05-1.23)** |
| Low social network | No | 133 | 29 (21.8) | Reference | Reference |  | 133 | 66 (49.6) | Reference | Reference |
|  | Yes | 55 | 8 (14.5) | 0.62 (0.26-1.47) | 0.50 (0.20-1.28) |  | 55 | 20 (36.4) | 0.59 (0.30-1.18) | 0.53 (0.25-1.15) |

**Supplemental Table 7.** β-coefficients and 95% confidence intervals (CI) for the association between logarithmic care hours and cognitive impairment, no dementia (CIND)/dementia status.

| **Care use hours** | **CIND/dementia × time** | **n** | **Model 1^a^**  **β (95% CI)^c^** | **Model 2^b^**  **β (95% CI)^c^** |  |
| --- | --- | --- | --- | --- | --- |
| **CIND-control sample** | | | | | |
| Formal care hours | No CIND × time | 488 | Reference | Reference |  |
|  | CIND × time | 244 | 0.18 (-0.11, 0.47) | 0.17 (-0.12, 0.47) |  |
| Informal care hours | No CIND × time | 488 | Reference | Reference |  |
|  | CIND × time | 244 | **0.42 (0.08, 0.76)** | **0.40 (0.05, 0.74)** |  |
| **Dementia-control sample** | | | | | |
| Formal care hours | No dementia × time | 350 | Reference | Reference |  |
|  | Dementia × time | 175 | -0.23 (-0.72, 0.26) | -0.28 (-0.78, 0.23) |  |
| Informal care hours | No dementia × time | 350 | Reference | Reference |  |
|  | Dementia × time | 175 | **1.48 (0.98, 1.97)** | **1.39 (0.88, 1.91)** |  |

^a^ Adjusted for age, sex, and educational attainment.

^b^ Further adjusted for living alone, disease count, and social network.

^c^ β-coefficients representing interactions between cognitive status and follow-up time.

A small value, 1 minute per month, was added to non-care users to ensure the logarithm of dependent variables.

**Supplemental Figure 1.** Flowchart of the study population

**
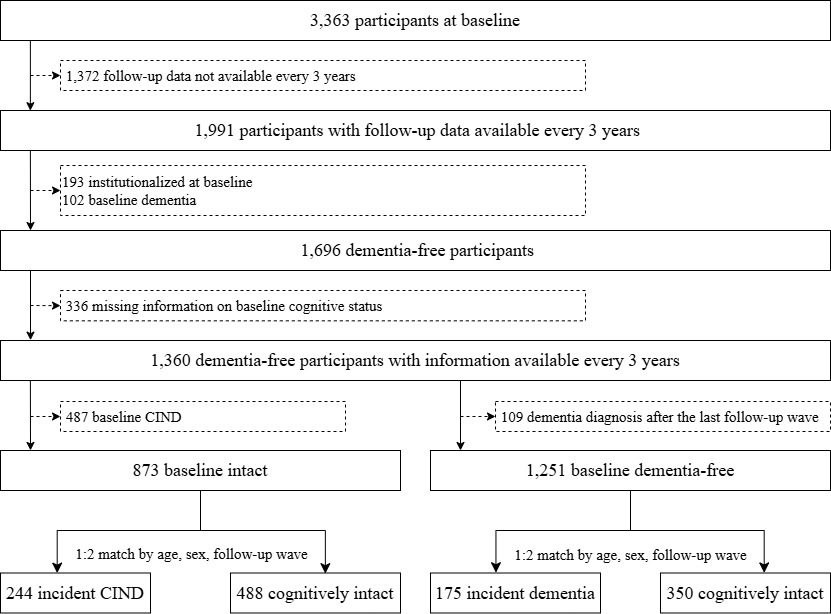
**

CIND: cognitive impairment, no dementia.
